# Hormone Replacement Therapy and Risk for Neurodegenerative Diseases

**DOI:** 10.1155/2012/258454

**Published:** 2012-04-04

**Authors:** Richelin V. Dye, Karen J. Miller, Elyse J. Singer, Andrew J. Levine

**Affiliations:** ^1^Longevity Center, Department of Psychiatry and Biobehavioral Sciences, David Geffen School of Medicine at the University of California, Los Angeles, CA 90025, USA; ^2^National Neurological AIDS Bank, Department of Neurology, David Geffen School of Medicine at the University of California, Los Angeles, CA 90025, USA

## Abstract

Over the past two decades, there has been a significant amount of research investigating the risks and benefits of hormone replacement therapy (HRT) with regards to neurodegenerative disease. Here, we review basic science studies, randomized clinical trials, and epidemiological studies, and discuss the putative neuroprotective effects of HRT in the context of Alzheimer's disease, Parkinson's disease, frontotemporal dementia, and HIV-associated neurocognitive disorder. Findings to date suggest a reduced risk of Alzheimer's disease and improved cognitive functioning of postmenopausal women who use 17*β*-estradiol. With regards to Parkinson's disease, there is consistent evidence from basic science studies for a neuroprotective effect of 17*β*-estradiol; however, results of clinical and epidemiological studies are inconclusive at this time, and there is a paucity of research examining the association between HRT and Parkinson's-related neurocognitive impairment. Even less understood are the effects of HRT on risk for frontotemporal dementia and HIV-associated neurocognitive disorder. Limits to the existing research are discussed, along with proposed future directions for the investigation of HRT and neurodegenerative diseases.

## 1. Introduction

Hormone replacement therapy (HRT), defined here as use of various types of estrogen alone or in conjunction with progestins (synthetic or exogenous progestogen), has long been studied as a possible prophylactic against Alzheimer's disease. While the association between HRT and Alzheimer's disease has been explored through several observational and randomized clinical trials to date, the relationship between HRT and other neurodegenerative diseases has received relatively little attention. In this review, we explore the body of research on HRT as a prophylactic against various neurodegenerative conditions, including Alzheimer's disease, Parkinson's disease, frontotemporal dementia, and HIV-associated neurocognitive disorder. In reviewing observational studies, randomized clinical trials, and basic science studies, we find evidence that some forms of HRT are neuroprotective, resulting in preservation of cognitive abilities in healthy postmenopausal women, improvement of Parkinson's symptoms, and variably altering risk of neurodegenerative disease.

## 2. Alzheimer's Disease

Alzheimer's disease (AD) represents the most common neurodegenerative disease, accounting for more than 50% of all dementia types [1]. Within the United States alone, national prevalence estimates indicate that AD affects 2.4 million individuals aged 70 and older [1, 2]. With increasing age, AD progressively affects more individuals, affecting 2.5% of those aged 71–79, 18% of those aged 80–89, and 30% of those aged 90 and older [1, 2]. 

Cognitive decline in AD is characterized by insidious onset and gradual progression over a course of several years [3–5]. Clinical research has identified subtle losses of cognitive functioning that precede AD. Studies have consistently shown that a deficit in episodic or verbal memory, specifically the ability to encode novel information, is an early symptom of AD and often presents several years before a formal diagnosis of AD [3, 6–9]. Such observations have led to the identification of a preclinical stage of AD that represents the transition between normal aging and AD. Specifically, mild cognitive impairment (MCI) represents the mild neurocognitive decline that occurs in the presence of relatively intact day-to-day functioning [4, 5]. Although there are several subtypes of MCI, the subtypes that are at increased risk for the development of AD involve predominant memory impairment. It has been estimated that approximately 10–15% of those diagnosed with MCI with predominant memory impairment convert to AD per year [4, 5]. The identification of MCI as a possible prodrome to AD, as well as the recent development classifier algorithms that assess later risk for AD based on a variety of clinical factors [10], leaves open the potential for initiating therapies, including HRT, that may prevent progression to AD in those at risk.

### 2.1. Estrogen and Risk for AD—Observational Studies

Incidence rates indicate the risk of AD among women is double that of men after the age of 80, even after controlling for protective factors such as education [11]. The higher incidence rate of AD among women have led to explorations on the association between estrogen deficiency and AD.

Observational studies have examined both HRT and estrogen replacement therapy (ERT), or estrogen alone, in relation to incidence of AD (see Table 1). For instance, in a sample of 514 women enrolled in the Baltimore Longitudinal Study of Aging, Kawas et al. found that ERT was associated with significantly reduced risk for AD [12]. Although the duration of use ranged between 1–15 years, the data did not show a significant effect for duration of ERT. In addition, no effect was observed for age of menopause. In another observational study reported by Tang et al., ERT was also associated with significantly reduced risk for AD in a sample of 1124 women enrolled in the Manhattan Study of Aging [13]. Here, however, an inverse relationship was observed for duration of use and risk for AD, with the lowest risk noted for women taking estrogen for longer than one year. Other observational studies have provided moderate support for decreased AD risk with ERT and the importance of duration of use. Using retrospective data on a sample of 355 women, Paganini-Hill and Henderson found that ERT was associated with moderately reduced risk for development of AD [14]. An inverse relationship was seen for duration of ERT and risk for all-cause dementia (AD as well as other causes of dementia), with those on ERT for seven or more years having the lowest risk for AD. While findings from these observational studies suggest that ERT may reduce risk of AD, given the nature of observational studies the findings may be affected by several biases. Specifically, the women who decided to take ERT for several years may have been healthier to begin with; they may have also been more proactive in seeking early postmenopausal treatment due to higher education and/or availability of resources. An additional criticism is the lack of controls in the studies; for instance, all observational studies described above involved varied ERT regimens among all participants rather than a uniform ERT regimen. Thus, the findings of the observational studies present with several limitations.

Although all of the studies examined above have included women who underwent natural menopause, recent observational studies have examined the differences between women who underwent natural versus surgical menopause [15]. In one, women who had surgical menopause demonstrated an increased long-term risk for cognitive impairment compared to women with natural menopause. In another paper based on the same data, the same researchers reported a linear trend, with increased risk seen with younger age at oophorectomy (bilateral or unilateral) [16]. These findings suggest that earlier age of surgical menopause increased risk of cognitive impairment and that estrogen deficiency may initiate risk for neurological diseases such as AD. Notably, the researchers also found increased risk of depression and cardiovascular disease among women with history of bilateral oopherectomy, suggesting that the relationship between surgical menopause and cognitive impairment may be multifactorial [17, 38].

### 2.2. Randomized Clinical Trials of HRT in Healthy and At-Risk Women

While observational studies generally support a neuroprotective role for ERT against AD, the results of randomized clinical trials (RCTs) have been equivocal. To date, the largest study has been the Women's Health Initiative Memory Study (WHIMS), an ancillary study of the Women's Health Initiative (WHI), a prospective study that enrolled 7479 postmenopausal women [39, 40]. A total of 4532 women with natural menopause (intact uterus) were randomized into a trial comparing conjugated equine estrogen (CEE) + medroxyprogesterone (MPA) versus placebo [40]. However, the trial was discontinued before completion due to unexpected health risks. Despite the early termination, data revealed that women who received CEE + MPA demonstrated greater cognitive decline compared to the placebo group [40]. Additional analyses revealed that risk for dementia was doubled for women who received CEE + MPA compared to the placebo group [39]. Taken together, data from the WHIMS demonstrated a higher incidence of dementia and greater cognitive decline among hormone users relative to placebo groups.

Although the WHIMS has been considered one of the largest and longest randomized studies examining HRT and cognitive deficits, generalizability of the findings is affected by several limitations. First, external validity of the WHIMS findings has come into question, as the participants in the treatment group were at high risk for cardiovascular and cerebrovascular disease; thus the higher rates of dementia may have been attributed to vascular disease. Second, in their analyses of the WHIMS data, the researchers included all dementia types into an “all-cause” dementia that included AD, vascular dementia, dementia due to Parkinson's disease, and frontotemporal dementia, thus limiting the interpretation of results. Third, a methodological limitation included the unavailability of baseline cognitive measures prior to treatment; thus, participants may have already been cognitively impaired prior to beginning HRT. Still another criticism has been the age of the participants; participants were age 65 or older, at least a decade past the average age of menopause. Together, these limitations have called into question the validity of the WHIMS findings, suggesting that the WHIMS may not be the best model for understanding the effect of HRT on Alzheimer's disease.

Another limitation in the generalizability of the WHIMS involves the type of HRT that was used. Specifically, it has been pointed out that CEE does not contain the hormone 17*β*-estradiol, [41] the estrogen compound that has been shown in basic science studies to be neuroprotective [42–44]. In addition, the greater rates of dementia seen among participants of the CEE + MPA study trial of the WHIMS suggest that simultaneous use of MPA may present additional risk [45]. Indeed, consistent with WHIMS findings, a recent randomized-controlled study by Maki et al. found that women receiving CEE + MPA for four months demonstrated mild declines in verbal memory compared to women receiving placebo [21]. Additionally, a recent comparison of several different HRT types has provided some insight into which treatment provides the most cognitive benefit. Using functional neuroimaging as an outcome measure, Silverman et al. compared the cerebral metabolic activity associated with three hormone regimens over the course of one year: 17*β*-estradiol (E2), CEE, and CEE + progestin [24]. Results revealed that the E2 group performed significantly better on verbal memory than the CEE group. This group also demonstrated higher metabolism in the receptive language and auditory association areas. Additionally, the CEE + progestin group demonstrated lower metabolism in areas associated with long-term memory storage (i.e., mesial and inferior lateral temporal regions) compared to the CEE group. Taken together, these findings suggest that E2-based therapies may provide the most beneficial neuroprotective effect. In addition, the Silverman et al. study suggests that combination therapies that include progestin may actually dampen the beneficial effects of estrogen.

Since the discontinuation of the WHIMS trials, the case for ERT in reducing the risk for AD and improving the cognitive functioning of postmenopausal women has continued to gain at least modest support through further RCTs. Indeed, results from several RCTs published in the past few years have demonstrated support that E2 formulations are associated with a reduced amount of decline in verbal memory among healthy postmenopausal women when compared to controls. The benefits of these treatments have been observed in trials with durations ranging from three months to two years (see Table 2) [18, 22–24]. In contrast, at least one study has found no benefit on verbal memory associated with E2 compared to placebo [20]; however, it was noted that the women in that study used E2 for only two months. Thus, it is possible that the effects of E2 on verbal memory may be evident only after three months or more. In a separate study, Joffe et al. found that E2 was not associated with an improvement in verbal memory scores but rather decreased likelihood for errors during the memory tasks [19]. Specifically, women on E2 demonstrated less perseverative errors during recall tasks compared to women on placebo. These women, as a group, were also less likely to demonstrate an interference effect when retaining previously learned information. Thus, although E2 was not found to enhance verbal memory scores per se, the authors concluded that E2 enhanced verbal information processing by decreasing the forgetfulness of a response already given [19]. 

### 2.3. Neuropathological and Neurophysiological Studies of HRT: Relevance to AD

While results of recent RCTs show modest support for a beneficial effect, evidence from histopathological and neurophysiological studies has provided stronger support for estrogen's neuroprotective effects, particularly for the neurodegenerative disease process thought to underlie AD [46–48]. Neuroimaging and autopsy results have indicated that *β*-amyloid and tau proteins are involved in the structural changes that lead to AD pathology, particularly in the hippocampus and other medial temporal regions, as well as the parietal and frontal cortical regions [49]. Evidence has shown that estrogen (particularly E2) provides protection against *β*-amyloid-induced damage and tau-related changes [50]. Observational and RCT studies that also utilized neuroimaging outcomes have also been supportive of the benefits of 17*β*-estradiol, particularly in the brain regions that show preclinical abnormalities in individuals who are at risk for AD. For instance, as mentioned earlier, E2 has been associated with higher metabolism in language processing and auditory association areas compared to other HRT regimens (CEE or CEE + MPA) [24]. However, observational studies and RCTs have also demonstrated support for varied ERT regimens. Compared to nonusers, long-term ERT (E2 or CEE for an average of 15 years) has been associated with increased cerebral blood flow to the hippocampus and left superior temporal gyrus at a two-year followup [51]. Further, compared to placebo, a four-month trial of ethinylestradiol and progestin was associated with increased activation in brain regions associated with the left middle/superior frontal cortex, and left inferior parietal cortex during verbal memory encoding tasks on functional magnetic resonance imaging [52]. Finally, in another study, long-term users of ERT (E2 or CEE for an average of 18 years) demonstrated higher density of muscarinic receptors in the hippocampus and prefrontal cortex than individuals who had never used ERT, suggesting that one of the neuroprotective effects of E2 or other ERT regimens could also include the maintenance of the cholinergic system in the hippocampus and frontal cortex [48].

A recently proposed explanation may explain the inconsistent results of the aforementioned observational studies and RCTs. Known as the “healthy-cell bias” [53], the hypothesis is that E2 may selectively benefit healthy neurons. In the context of human studies, based on the findings from observational studies and RCTs, this hypothesis predicts that E2 can be protective if initiated before or during times of neuronal stress, but harmful if given after the cells have progressed toward degeneration. In their study, Chen et al. administered E2 to rat hippocampal neurons exposed to *β*-amyloid, using varied doses and dose schedules (acute versus continuous versus intermittent). Data indicated that neurodegeneration was prevented when E2 was administered before or during *β*-amyloid exposure, and a continuous dose was found to demonstrate the strongest effects. In contrast, exposure to higher doses of E2 actually worsened neuronal death when *β*-amyloid was present. Additionally, E2 administered after *β*-amyloid exposure exacerbated neuronal death. It was concluded that the best E2 dosing was pretreatment and continuous exposure to prevent degeneration. Consistent with the “healthy-cell bias” hypothesis, Dumas et al. demonstrated a selective benefit of 17*β*-estradiol toward cognitively intact women [22]. A group of 142 postmenopausal women (age range: 61–87) were randomized to receive E2 (*n* = 70) or placebo (*n* = 72) for two years. Verbal memory was assessed at baseline and at 1-year and 2-year followup. Results revealed that women who received E2 and who performed at or above average on verbal recall at baseline demonstrated higher scores at the 1-year and 2-year followup compared to the placebo group. In contrast, women who received E2 and performed below average on verbal recall at baseline showed no significant difference compared to the placebo group. Dumas et al. concluded that these findings provided support to the healthy cell bias hypothesis, as they considered it improbable that women with a normal score or better had significant neurodegenerative changes [22]. Notably, the women who benefitted from estrogen exposure were age 70 (average) and approximately 20 years postmenopause, suggesting that older women who have intact verbal memory can benefit from a new regimen of ERT late in life, as long as they have not demonstrated memory impairment. Interestingly, basic science research has supported the biased neuroprotective effect of E2 toward healthy individuals; in fact, the presence of apoplipoprotein E4 (APOE4) genotype has been found to reduce the neuroprotective role of E2 in an animal model [54]. Thus, an alternative explanation for the findings of Dumas et al. could be that the women who demonstrated lower than average recall at baseline may have had the APOE4 genotype; in turn, they may have not experienced the neuroprotective effects of ERT. The healthy cell bias hypothesis also helps explain the finding, reported in most observational studies, of an inverse relationship between length of HRT treatment and risk for AD. 

Other investigators have hypothesized that there may be a “critical period” for postmenopausal women during which 17*β*-estradiol selectively provides a beneficial effect for younger as opposed to older women with an intact uterus [41, 55]. This hypothesis has also received support from at least one RCT. For example, LeBlanc et al. randomized 22 postmenopausal women to receive either E2 or placebo for 3 months [20]. At the end of the trial, the antimuscarinic drug scopolamine (SCOP) was administered before a verbal task to initiate anticholinergic-induced memory impairment. Results showed that E2 pretreatment significantly decreased the anticholinergic-induced impairment on the verbal memory task for the younger group (age 50–62); however, the benefit of E2 was not observed in the older group (age 70–81). Interestingly, the beneficial effects of E2 were only observed during the anticholinergic challenge with SCOP and not during the placebo challenge. LeBlanc et al. concluded that younger women benefit from E2 more than older women, and that the benefits of E2 in younger women may be observed only when the cholinergic system is temporarily disrupted. Consistent with this finding is that younger women have a higher density of muscarinic receptors than older women, and thus may be more sensitive to cholinergic changes [48]. Thus, it is plausible that the women in the aforementioned WHIMS may have been past the “critical period” for the beneficial effects of E2.

### 2.4. Summary—AD

Taken together, the findings from studies employing a variety of methods demonstrate that some forms of ERT are neuroprotective, resulting in preservation of cognitive abilities and reduced risk of AD. While some studies have affirmed that young and healthy postmenopausal women may benefit the most from estrogen exposure, other studies have suggested that older and healthy women with intact verbal memory can also benefit from estrogen. The consistent findings from the observational studies reviewed above seem to be that ERT (most commonly CEE), with a minimal duration of at least one year, is beneficial in reducing risk for AD among healthy postmenopausal women. Although benefits have been observed among varied regimens (CEE, CEE + P, E2) [48, 51, 52]; the most beneficial estrogen formulation seems to be E2 unopposed by progestin [24, 50]. Randomized clinical trials in healthy, postmenopausal women have suggested that E2 has been most beneficial in reducing cognitive decline, particularly verbal memory, which is the predominant symptom of early AD [18, 22–24]. Additionally, both observational and RCT studies utilizing neuroimaging outcomes have been supportive of the benefits of E2, particularly in the brain regions that show preclinical abnormalities in individuals who are at risk for AD [21, 24, 51, 52].

## 3. Parkinson's Disease

Parkinson's disease (PD) is the second most common neurodegenerative disorder after AD, with an estimated prevalence of 0.3% in the general population. Risk increases with age, with a prevalence of 1% in those over 60, and 4% in those 80 years and older [56]. Many, but not all, studies have reported higher risk for PD and younger age of onset in males [57–66]. This observation, along with the fact that the neuropathological process underlying PD commonly begins before menopause, suggests that estrogen may play a modulatory role. In addition, estrogen has a direct modulatory affects on dopaminergic functioning [67]. Together, these observations suggest a potential protective effect of estrogen against PD, or ameliorative impact on symptoms.

### 3.1. Estrogen and PD Symptoms

A variety of studies have addressed the impact of estrogen on PD. Perhaps the most indirect are observational studies of PD symptoms during the menstrual cycle. Early studies in the 1980s reported that some female patients with PD had fluctuations in motor symptoms that paralleled presumed fluctuations in endogenous estrogen levels [68, 69], with presumably lower levels of estrogen associated with greater motor symptoms. However, more recent studies have shown mixed results. Kompoliti et al. did not find significant correlation between endogenous hormone levels and motor examination in the “off” state (a state of decreased mobility as a result of nonresponsiveness to medication) among female PD patients examined at various times during their menstrual cycle [70].

A small number of prospective studies of ERT and PD have also been reported, with mixed results (Table 4). Strijks et al. did not find a significant dopaminergic effect in their 8-week placebo-controlled, randomized, double-blind trial pilot study of E2 in 12 postmenopausal female patients under the age of 80 [35]. However, an 8-week double-blind, parallel-group, prospective study using Premarin (CEE) versus placebo in PD patients with motor fluctuations showed a statistically significant improvement in “off" times (i.e., when dopamine agonist medications have diminished efficacy) among the estrogen treated group [36]. Further, another double-blind, placebo-controlled crossover study of high-dose transdermal E2 in 8 postmenopausal women with mild-to-moderate PD demonstrated a slight anti-Parkinsonian effect without significantly worsening dyskinesias [34].

Although the overall symptomatic effect of ERT on PD remains unclear, these early studies raised the possibility that some forms of estrogen may mitigate the symptoms of PD. Despite this early optimism, a more recent multicenter, randomized, double-blind, placebo-controlled, pilot trial of CEE in postmenopausal women with PD experiencing motor fluctuations did not find any benefit of ERT in ameliorating symptoms [37]. In that study, 23 women received either 0.625 mg/day of CEE or matching placebo for 8 weeks. None of the outcome measures, including changes from baseline to study completion in Unified PD Rating Scale scores, “on” time (i.e., duration that dopamine agonist medication is effective), dyskinesia ratings, and results from neuropsychological testing, were significantly different between the placebo and treatment groups, although the authors emphasized a nonsignificant trend of improvement on the total and motor scores of the Unified PD Rating Scale. It is conceivable that the null findings were due to the small sample size; however, the existing literature on ERT and PD symptoms remains equivocal at this time.

### 3.2. HRT and Observational Studies of PD Risk

Epidemiological studies of the protective effects of HRT against PD have been mixed as well (Table 3). The relationship between lifetime reproductive events and PD was examined by Martignoni et al. Comparing a large sample of women diagnosed with PD to healthy controls, they found that the duration of reproductive life was similar between the two groups [28]. Time and mode of menopause onset were also similar between the groups; however, women with PD reported less access to HRT. In addition, the PD group overall reported more premenstrual symptoms, fewer deliveries and abortions, and less use of contraception, indicating a relationship between PD and reproductive events. Benedetti et al. reported a case-control study in which women with PD had an earlier reported age of menopause, a higher frequency of hysterectomies, and lower occurrence of HRT [27]. Further, Currie et al. found that ERT in postmenopausal women was associated with a significantly reduced risk of developing PD [29], and Ragonese et al. found that factors reducing estrogen stimulation during life were associated with development of PD [30]. Specifically, PD was significantly associated with shorter fertile life lengths (<36 years) and a longer cumulative length of pregnancies (>30 months). This group later reported a significant correlation between age of PD onset and both age at menopause and fertile life duration [32]. Despite these findings, others have found contrary results. Popat et al. found that the association of postmenopausal HRT and PD risk depended on the type of menopause [31]. Among women with history of hysterectomy (with or without an oopherectomy), ERT use was associated with a 2.6-fold increased risk for PD, and a trend for additional risk was noted for increasing duration of estrogen use. Conversely, among women with natural menopause, no increased risk of PD was observed with HRT (ERT alone or in conjunction with progestin). Contrary to the findings of Benedetti et al., earlier age of menopause was associated with reduced risk of PD. Further, Simon et al. recently reported results of a 22-year prospective study of 244 participants in the Nurses' Health Study who developed PD [33]. Among their sample, risk of PD was not significantly associated with reproductive factors or HRT use. However, they did find that use of HRT may modify the associations of smoking and caffeine with PD risk; specifically, the inverse relationship between caffeine use and risk of PD was observed only in non-HRT users. Further, whereas the researchers also reported an inverse relationship between pack-years of smoking and risk of PD for both HRT users and nonusers, risk was reduced more in the latter group. As such, HRT use appeared to attenuate the observed beneficial effects of caffeine use and tobacco smoking. Of note, this study did not separately analyze the data based on type of HRT.

In one of the largest observational studies to date, Rocca et al. examined 1,252 women with unilateral oophorectomy, 1,075 women with bilateral oophorectomy, and 2,368 controls for development of PD. Data for the participants were collected until death or the termination of the study using direct or proxy interviews, neurologic examinations, medical records, and/or death certificates. The authors found that women who underwent either unilateral or bilateral oopherectomy before the onset of natural menopause, thereby decreasing endogenous estrogen levels, had an increased risk of parkinsonism compared with referent women. Further, risk increased with younger age at oophorectomy. The findings were similar regardless of unilateral or bilateral oopherectomy. Importantly, while the authors reported a trend, the surgical menopause group was not at increased risk for PD.

Although these studies might appear to provide conflicting results, complex factors are at play. The indication for HRT (posthysterectomy, posthysterectomy + oopherectomy, natural menopause), the specific type of HRT (CEE, E2, estrogen/progestin combinations), and other variables may combine in ways yet unknown to increase or decrease PD risk. Clearly, further study is necessary.

### 3.3. Studies of HRT and Dementia due to PD

PD is also associated with cognitive decline, with anywhere between 24–31% becoming demented [71]. PD dementia is considered a subcortical dementia, with associated deficits ranging from simple motor ability to higher-order cognitive functions [72]. Despite the high incidence of neurocognitive dysfunction in PD, the relationship between HRT and dementia in those with Parkinson's disease has received considerably less attention. Only two case-control studies were found. Marder and colleagues investigated risk of PD both with and without dementia among a sample of 1156 women. They reported that ERT protected against development of PD-associated dementia, but not against PD itself [25]. Similarly, Fernandez and Lapane found that estrogen use was associated with better cognitive functioning and greater independence in activities of daily living among a large sample of elderly women living in nursing homes [26]. They also noted that estrogen users were more depressed and likely to be on an antidepressant as compared to nonusers. One-year death rates were comparable between estrogen users and nonusers.

### 3.4. Mechanisms of Estrogen Action in PD

While epidemiologic, observational, and experimental studies of ERT and PD have produced equivocal results, the biological mechanisms for a beneficial effect of estrogen upon dopaminergic functioning are less so. There are two general mechanisms of action through which estrogen might influence PD: symptomatic and neuroprotective. Estrogen receptors have been located in the nuclei of nigral dopaminergic (DA) neurons, including estrogen receptor alpha (ER*α*) and beta (ER*β*) [73, 74], suggesting that estrogen might therefore directly influence DA functioning. ER*α* has also been found in midbrain glial cells [75], and ER*β* in striatal medium spiny neurons [74]. Novel surface membrane estrogen receptors have also been described [76, 77]. Perhaps related to these, administration of exogenous conjugated estrogens results in an increase in binding of the DA transporter ligand TRODAT in otherwise healthy postmenopausal women [78]. It has also been shown that, in the absence of nigral neuroprotection, central E2 synthesis limits striatal DA loss caused by 6-OHDA in male rodents, implicating a modulatory effect on DA function [79]. These studies provide evidence that estrogens may upregulate the nigrostriatal pathway, either pre- or postsynaptically, by an effect on nuclear or surface membrane estrogen receptors.

Estrogen's neuroprotective actions have been well established. In PD, there are animal models that are exquisitely specific for nigral cell death, of which the 6-hydroxydopamine (6-OHDA) and MPTP/MPP+ models are perhaps the best known [80, 81]. There is ample evidence that both endogenous and exogenous estrogen ameliorate DA depletion in the MPTP/MPP+ model [75, 82–91]. There is similar evidence that estrogen is neuroprotective in the 6-OHDA animal model [79, 92–96], a methamphetamine model [97–100], and a wide range of other relevant animal models [101–103]. The exact mechanisms of neuroprotection, however, are not clear. Studies have shown a role for binding of estrogen to the nuclear estrogen receptor [104], the ER*α* subtype, [105] ER*α* with a glial contribution, [75] ER*α* + ER*β* [106], and ER-independent mechanisms [88]. This has implications for potential therapeutic agents, as some estrogen analogues lack activity at one or both nuclear receptors; while others, such as the “inactive” enantiomer E2, may have no ER binding activity at all. E2 has been shown in the MPTP model to have neuroprotective properties [101], and has been investigated as a possible neuroprotective agent [107].

It is important, however, to recognize the imperfect nature of these preclinical models. First, while PD is a chronic, slowly progressive disorder, the aforementioned animal models use agents that cause acute toxicity. Second, despite the wide use of these models over the past two decades and the demonstration in preclinical models that many agents are neuroprotective against 6-OHDA, MPTP, or both, none of these agents have proven neuroprotective in human subjects with PD. There may be a simple explanation for this. We now know that neurodegeneration in most cases of familial PD is due to impaired ubiquitin-proteosomal function and alpha-synuclein protein aggregation [108]. Although the relationship between these abnormalities and those replicated by the 6-OHDA and MPTP models are complex, it appears likely that any agent that will be neuroprotective in humans with idiopathic PD will need to act to reduce alpha-synuclein aggregation. This can occur either by reducing its synthesis, reducing protein aggregation, enhancing its elimination, or reducing the toxic effects of excessive alpha-synuclein. Only recently has evidence been found that estrogen has the ability to act on alpha-synuclein in a beneficial manner. Hirohata et al. found a variety of sex hormones, including estriol, estradiol, estrone, androstenedione, and testosterone to exert significant antiaggregation and fibril-destabilizing effects on alpha-synuclein* in vitro*. Estradiol was especially effective [109]. Further, Marwarha et al. showed that activation of ER*β*, in conjunction with inhibition of LXR*β*, may reduce progression of PD by slowing *α*-synuclein accumulation.

### 3.5. Summary—PD

While *in vitro* and non-human* in vivo* experiments have consistently demonstrated evidence for estradiol's neuroprotective activity in dopaminergic neurons and animal models of PD, results of clinical and epidemiological studies are inconclusive at this time. Recent findings of estradiol's modulation of alpha-synuclein indicate a specific mechanism through which the hormone may reduce risk for PD and/or mitigate symptoms. Longer clinical trials with specific estrogen compounds (i.e., 17*β*-estradiol), as well as biological markers of disease progress (e.g., neuroimaging), will be more likely to definitively determine if ERT is protective against PD or if it can mitigate the disease. With specific regards to PD-associated dementia, only two case-control studies were located, both suggesting that ERT reduces risk of cognitive impairment in women with PD.

## 4. HIV-Associated Neurocognitive Disorder (HAND)

Internationally, an estimated 33 million individuals have HIV/AIDS, [110] and in many areas women comprise the majority of those infected [111]. Aggressive intervention with a regimen of multiple antiretroviral drugs (combined antiretroviral therapy, or cART) has successfully increased lifespan and attenuated some of the most dire neurological effects of HIV infection. However, cART cannot eradicate HIV, and it has attenuated, not eliminated, the most common neurological complication of HIV, or HIV-associated neurocognitive disorder (HAND) [112]. In this section, we discuss what is known about estrogen and HAND from observational studies in humans, studies in animal models, and *in vitro* studies. No relevant human clinical trials of estrogen for HAND have been published.

HAND is a constellation of cognitive impairments caused by HIV infection [112]. Because of the lack of diagnostic biomarkers, HAND remains largely a clinical diagnosis, made when an HIV+ individual experiences neurocognitive decline, sometimes with concomitant deficits in day-to-day functioning, and only after other conditions that might cause this decline have been ruled out. The severity of HAND ranges between mild neurocognitive impairment with no impact on day-to-day functioning to a debilitating HIV-associated dementia [112]. While the incidence of new cases of HAD has declined dramatically [113, 114], the prevalence of milder forms of HAND has actually increased along with the longevity of the cART-treated HIV+ population [113]. This phenomenon has been variously ascribed to several explanations, including the presence of irreparable CNS damage pre-cART [115], the failure of many cART regimens to adequately penetrate and treat the CNS [116], persistent low levels of HIV despite treatment [117], and to persistent CNS inflammation [118], among others. The latter is particularly relevant to the putative therapeutic benefit of estrogen, as it appears that cART does not always reduce and in some cases may increase, the CNS inflammation [119] that is associated with HAND [120]. Estrogen has significant anti-inflammatory and neuroprotective properties [121–123] and can potentially counteract inflammation in the HIV+ brain, as discussed in more detail below.

There are several other important reasons for investigating the use of estrogen as an adjunctive treatment in HIV and HAND. First, estrogen and other gonadal steroids have significant effects on the course and presentation of HIV disease itself. For example, women are at increased risk for acquiring HIV compared to men, and this vulnerability may be affected by gonadal hormones [124]. Further, in a macaque model of HIV infection, progestogen-based hormonal contraceptives increased the risk of acquiring simian immunodeficiency virus (SIV), increased disease progression, and increased genital shedding of SIV; whereas treatment with estrogen lowered risk of acquiring SIV [125]. Results of natural history studies suggest a gender role in disease progression, possibly due to hormonal differences. For example, women have lower HIV RNA viral loads at seroconversion compared to men [126], and when adjusted for CD4+ count, women have lower viral loads throughout the course of their infection [127]. While one study found a lower risk of clinical progression to AIDS among HIV+ women versus HIV+ men treated with cART [128], others have found no differences in clinical outcome by gender [129]. A possible explanation for such gender disparity, should it turn out to be valid, is estrogen, which decreases HIV replication in peripheral blood mononuclear cells [130]. However, all such studies must be interpreted with caution because of the reported gender differences between HIV+ men and women in socioeconomic status, risk behavior, substance abuse, and access to care [131], which also affect progression to AIDS [132, 133]. With regards to HAND, whether women develop HAND at the same rate as men or if there are different clinical manifestations of HAND in men and women remains a controversial topic. In part, this is because so few studies had sufficient numbers of females to evaluate. A sub-study of the Women's Interagency HIV Study is beginning to address this problem [134].

There is neurobiological reason to expect a reduction of HIV-related neuropathological changes with ERT. Firstly, microglia are the resident immune cells of the CNS, and these cells play an important role in driving inflammation in many neurodegenerative diseases, thus representing an important target for therapy [135]. In HIV infection, microglia can be infected and/or activated; they are major sources of complete HIV virions, individual neurotoxic viral proteins, proinflammatory substances, and other potential mechanisms that drive neurotoxicity, neuroinflammation, oxidative stress, and neurodegeneration. Microglia express endogenous estrogen receptors [136], and treatment with estrogen is anti-inflammatory provided it is administered early in the course of an insult [121, 123]. Secondly, estrogen's anti-inflammatory effects may directly counteract the neuroinflammation caused by HIV proteins. HIV-infected cells can generate both replication-competent virions and excess viral proteins, which are shed or secreted into the extracellular space. The HIV coat protein, *gp120*, is the binding protein for viral entry [137] and acts as an indirect neurotoxin via its effects on microglia, macrophages, and astrocytes, initiating a cascade of events that damage neurons. Estrogen has been reported to have a broad anti-inflammatory effect on microglia [121]. Estrogen reduces the neuroinflammatory responses to gp120 and exerts neuroprotective effects on gp120-exposed neurons, by raising the levels of neurotrophins, decreasing apoptotic factors, and antioxidant properties [138]. Zemlyak et al. reported two different beneficial effects of estrogen in the amelioration of gp120-induced toxicity: a major effect of attenuating the neurotoxicity of factors released by gp120-treated microglial cultures, and a minor effect of enhancing the ability of neuronal cultures to survive exposure to neurotoxic factors [122]. Another neurotoxic HIV protein, *tat*, the nuclear trans-activating protein, is essential in promoting the transcription and replication of HIV. *tat* can act both directly to harm neurons [139], and indirectly by stimulating macrophages, microglia, and astrocytes to synthesize harmful substances such as proinflammatory cytokines [140], and by increasing free radicals and oxidative stress [141]. In cell culture, 17*β*-estradiol suppressed *tat*-activated transcription of HIV in astrocytes [142]. 17*β*-estradiol also attenuated the *tat*-induced release of pro-inflammatory mediators in endothelial cells [143], prevented oxidative stress and cell death associated with combined gp120 and *tat* neurotoxicity *in vitro *[144], and prevented gp120/*tat*-induced loss of dopamine transporter function [144].

These observations have led to the proposal that serum estradiol levels be maintained in HIV+ women as a possible neuroprotective agent against HAND [145]. Despite this, there is little clinical information about estrogen and HAND in HIV+ women. A single retrospective study from the pre-cART era, of 84 older (age 40+ years) HIV+ women, reported that hormone replacement therapy (HRT) was associated with a significantly decreased risk of mortality [146]. Of interest, there were six women in the cohort who were diagnosed with HIV-associated dementia, none of whom reported taking HRT. This study has been interpreted by some to indicate a neuroprotective effect of HRT; however, this was not a prospective study that examined cognition in an organized or standardized fashion. However, based on this last report and on the neuroprotective role of estrogen in other inflammatory and degenerative conditions, the role of estrogen and other hormones in HAND has become an area of growing interest among basic scientists.

No studies of HAND or neurocognitive functioning in HIV+ persons have considered hormonal status or use of exogenous hormones. The preponderance of evidence to date indicates that HIV+ men and women develop neurocognitive impairment at a similar rate, when issues such as access to care, education, and substance abuse history are similar. While some have reported a higher occurrence of HIV-associated dementia among women [147], others have not found this [148, 149]. More recently, Martin et al. studied a large well-matched group of adult male and females, stratified by HIV status, all with a history of substance dependence [150]. Participants were abstinent at the time of testing. Whereas the performance of HIV+ men did not differ from HIV-negative counterparts of measures of motor skill and probabilistic learning, the HIV+ women performed worse than their seronegative counterparts, suggesting that women might be more vulnerable to the effects of HIV. However, due to the absence of a nonsubstance-dependent control group, they could not exclude the possibility that the observed differences were due to gender-related differences in the cognitive effects of addiction. Another study reported no gender difference in rate of neurocognitive decline over time [151]; and still another found that while rates of impairment were similar between men and women, there were some differences in the neurocognitive profiles [148]. Whether this is related to estrogen or other gonadal hormones remains to be determined.

### 4.1. Summary—HIV/HAND

 HAND shares many features with other neurodegenerative diseases, including microglial activation and neuroinflammation. Preliminary studies in animal and *in vitro* models indicate that, like many other neurodegenerative diseases, the effect of HIV on the brain may be blunted by treatment with 17*β*-estradiol, and possibly other gonadotrophic hormones. This would have to be balanced against the risks of adding estrogen to the regimens of HIV+ patients, both male and female. However, there is a pressing need to determine if HRT may benefit patients with AIDS who remain at risk for HAND even when treated with HAART.

## 5. Frontotemporal Dementia

Frontotemporal dementia, or FTD, is the most common form of a group of related neurodegenerative diseases that primarily affect the frontal and/or temporal lobes. The others include semantic dementia and progressive nonfluent aphasia. Collectively, these have been called frontotemporal lobar degenerative diseases [152], and they are believed to account for an estimated 20% of dementia cases with presenile onset [153].

Only one study to date has addressed the relationship between HRT and FTD. Levine and Hewett reviewed the medical files of all women seen at an Alzheimer's disease center (ADC) in Central California and found that 70% of women diagnosed with FTD had been taking HRT (exact regimen unspecified) when evaluated, as compared to an estimated 24% of the surrounding population [154]. While one easy interpretation would be that women exhibiting cognitive impairment would have been more likely to be placed on HRT before coming to the ADC, only 20% of women diagnosed with AD at the same center had been taking HRT, so it is therefore unlikely that HRT was administered as a result of preclinical cognitive problems. The women diagnosed with FTD were also similar in age to women entering the center with AD (average age of symptom onset was 65, average age of initial evaluation was 70). While poor diagnostic accuracy and estrogen's beneficial effects on mood were cited as possible reasons for the findings, a more compelling reason offered was a marked upregulation of tau in response to E2 administration, as evidenced *in vitro *[155]. The neuropathological correlates of many FTD cases appear to be tau-related, and in some cases directly linked to mutations in the tau gene [156]. In such cases, E2 may increase risk of FTD by increasing production of mutated forms of tau. However, while the role of tau in FTD has been well established, it is now known that it does not account for all forms of FTD [157]. Still the relationship between tau and E2 is a compelling reason to further study the influence of ERT on risk for FTD.

## 6. Summary and Conclusions

**Figure 1:**
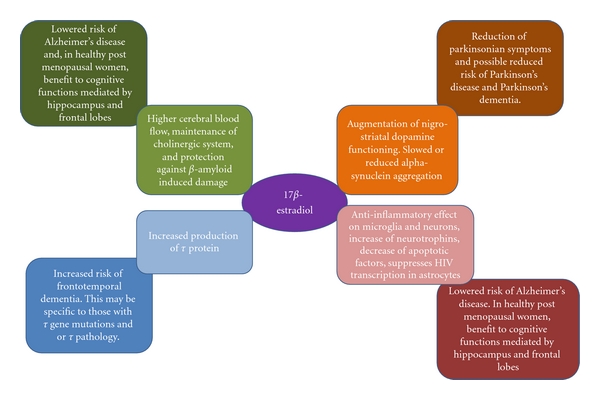
Putative mechanisms through which 17 *β*-estradiol exerts neuroprotective and neuro-adverse effects. In the context of Alzheimer's disease, Parkinson's disease, and HIV, 17*β*-estradiol appears to be neuroprotective. However, frontotemporal dementia is often the result of mutated tau protein and/or tau-related pathology. Because 17*β*-estradiol increases production of tau, it may accelerate risk for some forms of frontotemporal dementia.

In summarizing the evidence discussed above, HRT, in particular ERT, appears to play an efficacious role in treating and preventing several neurodegenerative conditions.  Figure 1 depicts putative neurobiological and neurobehaivoral sequelae resulting from 17*β*-estradiol use, based on studies reviewed in this paper. The case for a neuroprotective role of HRT and AD is supported by research from epidemiological and RCT studies, which have shown that estrogen, specifically E2 (17*β*-estradiol), can reduce the risk for AD and minimize cognitive decline in otherwise healthy women, particularly verbal memory. Based on basic science research, the mechanisms for this neuroprotection may involve E2's protection against *β*-amyloid-induced degeneration and may even include the maintenance of the cholinergic system in the hippocampus and frontal cortex. In addition, at least one study has demonstrated that the presence of progestins in combination therapies may actually dampen the beneficial effects of estrogen [24]. 

Similarly, *in vitro* and non-human *in vivo* experiments have demonstrated E2's neuroprotective effects in dopaminergic neurons and animal models of PD. In addition, E2's modulation of alpha-synuclein indicates a specific mechanism through which the hormone may reduce risk for PD and/or mitigate symptoms. To date, results of clinical and epidemiological studies of ERT alleviating motor symptoms in PD patients have been mixed and warrant further investigation. The effects of HRT on the neurocognitive symptoms of PD have received little attention, with the two case-control studies to date indicating that ERT reduces risk of cognitive impairment in women with PD.

Preliminary studies in animal and *in vitro* models indicate that treatment with E2, and possibly other gonadotrophic hormones, may reduce the effect of HIV on the brain. To date, much research on the neuroprotective effects for HIV neurodegenerative changes has been conducted on animal models and has yet to extend to humans. Nonetheless, preliminary research has suggested that development of HAND may be alleviated by HRT pretreatment. Conversely, and contrary to the findings from other neurodegenerative diseases, there is some evidence that E2 may actually augment risk for FTD via its action on tau.

Additional research is needed to further delineate the molecular mechanisms through which E2 and other estrogens act to delay or prevent neuropathological progression, or possibly cause progression in the case of FTD. Large-scale observational studies that accurately document HRT regimen and control for factors such as depression, education, and medical comorbidities (e.g., vascular risk factors) will also help to elucidate the role of ERT in the neurodegenerative disease etiology. While observational studies and RCTs examining ERT and AD have demonstrated long-term beneficial effects of varied ERT regimens (E2 or CEE), future studies may include long-term followup (5–10 years) of E2-based therapies alone on cognitive measures and neuroimaging outcomes, as such would provide helpful information on the duration of the benefits of E2 following discontinuation.

 Notably, possible medical risks should be considered in study of HRT and neurocognitive functioning [45]. For instance, breast cancer is often a substantial concern that is linked with HRT. In fact, it is claimed that combined HRT with estrogen plus progestin is a cause for breast cancer. However, while followup analysis approximately three years after termination of the WHI study demonstrated an increased risk for “all-cause cancer” for participants in the CEE + MPA trial compared to the placebo group [158], the risk for breast cancer and other types of cancer did not differ between groups. Similarly, recent retrospective analyses of the WHI data found insufficient evidence that estrogen plus progestin increased risk of breast cancer [159]. Another study using the WHI data found that among women in the CEE + MPA trial, increased breast cancer risk was especially pronounced among women with breast tenderness [160]. In fact, new onset of breast tenderness after HRT initiation was associated with increased breast cancer risk among women assigned to the CEE + MPA trial, but not among women assigned to CEE-alone. In contrast, an additional followup analyses after the termination of the WHI data demonstrated that participants in the CEE-alone trial did not demonstrate increased risk for breast cancer [161]. Although the available information is insufficient at this time to support a clear link between HRT and increased risk for breast cancer, at least one study from the WHI has reported an increased risk of breast cancer among users of estrogen plus progestin with new onset of breast tenderness. This is an issue that requires continued investigation.

In clinical settings, the financial cost will need to be considered when recommending E2-based therapies for prevention of AD or other neurodegenerative diseases. Patients and their physicians will have to determine whether the potential cognitive benefit associated with E2 will outweigh the financial cost, as well as the above-mentioned medical risks.

## Figures and Tables

**Table 1:** Observational studies of ERT and risk for dementia.

Study (reference)	Sample description	Overall findings
Paganini-Hill and Henderson [14]	355 postmenopausal women (165 users; 190 nonusers) with a mean age of 86.5 years at death; retrospective data from the Leisure World, Laguna Hills cohort	ERT (not specified) for 1–7 years was associated with reduced risk for AD (OR: 0.67, CI 95% 0.38–1.17) compared to nonusers. Risk for AD decreased with longer duration of use.
Tang et al. [13]	1124 healthy postmenopausal women (156 users; 968 nonusers), with a mean age of 74.2, enrolled in the Manhattan Study of Aging	After controlling for age, education, and ethnicity, ERT (majority used CEE) for 6–8 years was associated with lower risk for AD (OR 0.50, 95% CI, 0.25–0.90) compared to nonusers. Risk for AD decreased with longer duration of use.
Kawas et al. [12]	514 healthy postmenopausal women (230-users; 242-non-users), with a mean age of 65.5, enrolled in the Baltimore Longitudinal Study of Aging	After controlling for education, ERT (not specified) for 1–10 years was associated with lower risk for AD (OR: 0.46, 95% CI, 0.21–0.99) compared to non-users. No effect was observed for duration of use.
Rocca et al., [15–17]	813 women with unilateral oophorectomy, 676 women with bilateral oophorectomy, and 1,472 women who did not undergo oophorectomy.	Women who underwent oophorectomy (unilateral or bilateral) before onset of menopause were at increased risk for cognitive impairment or dementia (OR: 1.46, 95% CI, 1.13–1.90) compared to women who did not undergo oophorectomy. Risk increased with younger age at oophorectomy.

**Table 2:** Randomized clinical trials of HRT and verbal memory.

Study (reference)	Hormone treatment used	Sample size	Age	Outcome measure	Overall findings
Bagger et al., [18]	E2 2 mg + varied progestins versus placebo for 2 years	261	54.1	Cognitive screening task	Followup study of women randomized 5, 10 and 15 years earlier to HRT or placebo during clinical trials. Logistic regression showed that for women who received HRT for 2-3 years, the relative risk for cognitive impairment was significant decreased by 64% compared to the never users. Long-term/current users of HT also demonstrated a decreased risk of 66% compared to the never users.
Joffe et al. [19]	E2 0.5 mg versus placebo for 12 months	52	40–60	Verbal memory; Functional MRI	Women on E2 had fewer perseverative errors during verbal recall when placebo-treated women. Women on E2 also showed greater retention of new information without interference.
LeBlanc et al., [20]	Estradiol 2 mg versus placebo for 2 months	32	53.26 (treatment) 52.08 (placebo)	Verbal memory	Women on estrogen therapy did not show higher cognitive performance on verbal memory tasks compared to women on placebo.
Maki et al., [21]	(CEE) + medroxyprogesterone acetate (MPA) versus placebo for 4 months	158	51.9 (treatment) 52.4 (placebo)	Verbal memory	Modest negative effects on verbal memory (short- and long-term recall) were found in the HRT versus placebo group.
Dumas et al. [22]	E2 2 mg versus placebo for 3 months	22	50-62 (younger) 70–81 (older)	Verbal memory	All women were administered the antimuscarinic drug scopaline (SCOP) or placebo. E2 pretreatment significantly decreased the anticholinergic drug-induced impairments on verbal memory task for the younger group only compared to the older group.
Tierney et al. [23]	E2 1 mg versus placebo for 2 years	142	61–87	Verbal memory	Women on E2 who scored at or above average showed less decline in delay verbal memory compared to women on placebo.
Silverman et al. [24]	17*β*-estradiol (E2) versus conjugated equine estrogen (CEE) versus CEE + P for 1 year	53	50–65	Verbal memory; FDG-PET	Women on E2 had significantly higher verbal memory than CEE and showed higher metabolism in Wernicke's and auditory association. E2 was also associated with higher metabolism in mesial and inferior lateral temporal regions and inferior frontal cortex compared to PE.

**Table 3:** Case-control and epidemiological studies of HRT and Parkinson's disease.

Study (reference)	Sample description	Overall findings
Marder et al. [25]	87 women with Parkinson's disease without dementia (PDND), 80 women with Parkinson's disease with dementia (PDD), and 989 nondemented healthy women.	ERT reduced risk of dementia among the PD-only sample (OR = 0.22, 95% CI: 0.05–1.0), and also when PDD patients were compared to healthy controls (OR = 0.24, 95% CI: 0.07–0.78). ERT did not affect the risk of PD.
Fernandez and Lapane [26]	Data from 10,145 elderly women with PD available via the Systematic Assessment in Geriatric drug use via Epidemiology (SAGE) database. Included 195 women with PD who received estrogen and 9950 who did not receive estrogen.	Independent of age, estrogen users had better cognitive functioning and were more independent with regards to activities of daily living. More estrogen users were depressed and likely to be taking antidepressant medications.
Benedetti et al. [27]	72 women with PD and 72 healthy women.	The PD group had undergone hysterectomy (with or without unilateral oophorectomy) more than the control group (OR = 3.36; 95% CI: 1.05–10.77). The PD group had more frequent occurrence of early menopause (< or = 46 years) (OR = 2.18; 95% CI: 0.88–5.39). The PD group used ERT for at least 6 months after menopause less frequently than the control group (14%; OR = 0.47; 95% CI = 0.12–1.85). The PD group did not have earlier menopause than the control group.
Martignoni et al. [28]	150 women with idiopathic PD and 300 healthy women, all postmenopausal.	Duration of reproductive life was similar between women with PD and those without PD. Women with PD reported less access to HRT. The PD group also reported more premenstrual symptoms, fewer deliveries and abortions, and less use of contraception, indicating a relationship between PD and reproductive events.
Currie et al. [29]	68 women with PD and 72 healthy women, all postmenopausal.	50% of women in the control group took ERT, as compared to 25% of women in the PD group. Women who had taken postmenopausal ERT were less likely to develop PD than those who had not (odds ratio, 0.40; 95% CI: 0.19–0.84). Among women with PD, postmenopausal ERT was not associated with age of onset.
Ragonese et al. [30]	131 women with idiopathic PD and 131 healthy women.	PD was significantly associated with a fertile life length of less than 36 years (OR 2.07; 95% CI: 1.00 to 4.30). PD was also associated with a cumulative pregnancy length of longer than 30 months (OR 2.19; 95% CI: 1.22 to 3.91). There was an inverse association between PD and surgical menopause (OR 0.30; 95% CI: 0.13 to 0.77).
Popat et al. [31]	178 women with PD and 189 healthy women.	Among women with history of hysterectomy (with or without an oopherectomy), ERT use was associated with a 2.6-fold increased risk for PD, and a trend for additional risk was noted for increasing duration of estrogen use. Among women with natural menopause, no increased risk of PD was observed with HRT (ERT alone or in conjunction with progestin). Earlier age of menopause was associated with reduced risk of PD.
Ragonese et al. [32]	145 women with PD.	A significant correlation was found between age at PD onset and age at menopause, and also between age at PD onset and fertile life duration.
Rocca et al. [16, 17]	1,252 women with unilateral and 1,075 women with bilateral ophorectomy, and 2,368 referent women.	Women who underwent either unilateral or bilateral oophorectomy had an increased risk of parkinsonism compared to referent women (HR 1.68; 95% CI: 1.06–2.67). This risk increased with younger age at oophorectomy.
Simon et al. [33]	22-year prospective study of 244 women with PD enrolled in the Nurses' Health Study.	Risk of PD was not significantly associated with reproductive factors or HRT. The association of smoking and caffeine with PD risk was modified by HRT, however. Based on a very small sample (4), women using progestin only hormones had increased risk for PD.

**Table 4:** RCTs of ERT and Parkinson's disease.

Study (reference)	Hormone treatment used	Sample Size	Outcome measure	Overall findings
Blanchet [34]	High-dose transdermal E2. Cross-over design with 2 weeks on E2, 2 week washout, and 2 weeks on placebo	8	Therapeutic threshold for levodopa.	All but one participant had levodopa-induced dyskinesia at start of study. After 10 days of E2 treatment a significant reduction was observed in the anti-parkinsonian threshold dose of intravenous levodopa without significantly worsening dyskinesias.
Strijks et al. [35]	17*β*-estradiol (E2) versus placebo for 8 weeks	12	Motor score from the Unified Parkinson's Disease Rating Scale (UPDRS); patient report of subjective changes.	No differences in outcome measures between E2 and placebo.
Tsang et al. [36]	CEE versus placebo for 8 weeks	40	UPDRS, timed tapping score, Hamilton Depression Scale, patient self-report.	“On” and “off” times, and motor score on the UPDRS improved with estrogen.
The Parkinson Study Group Poetry I Investigators [37]	CEE versus Placebo for 8 weeks	23	Primary outcome was ability to complete the trial. Other outcome measures included adverse events, UPDRS, “on” time, dyskinesia ratings, and neuropsychological functioning.	The estrogen group showed a trend for improvement on the total and motor UPDRS scores.
